# Neurogastronomy as a Tool for Evaluating Emotions and Visual Preferences of Selected Food Served in Different Ways

**DOI:** 10.3390/foods10020354

**Published:** 2021-02-07

**Authors:** Jakub Berčík, Johana Paluchová, Katarína Neomániová

**Affiliations:** Department of Marketing and Trade, Faculty of Economics and Management, Slovak University of Agriculture, 949 76 Nitra, Slovakia; jakub.bercik@uniag.sk (J.B.); johana.paluchova@uniag.sk (J.P.)

**Keywords:** gastronomy, consumption, neurogastronomy, air quality, emotions, waffles, visual design, smell, eye tracking, face reading

## Abstract

The appearance of food provides certain expectations regarding the harmonization of taste, delicacy, and overall quality, which subsequently affects not only the intake itself but also many other features of the behavior of customers of catering facilities. The main goal of this article is to find out what effect the visual design of food (waffles) prepared from the same ingredients and served in three different ways—a stone plate, street food style, and a white classic plate—has on the consumer’s preferences. In addition to the classic tablet assistance personal interview (TAPI) tools, biometric methods such as eye tracking and face reading were used in order to obtain unconscious feedback. During testing, air quality in the room by means of the Extech device and the influence of the visual design of food on the perception of its smell were checked. At the end of the paper, we point out the importance of using classical feedback collection techniques (TAPI) and their extension in measuring subconscious reactions based on monitoring the eye movements and facial expressions of the respondents, which provides a whole new perspective on the perception of visual design and serving food as well as more effective targeting and use of corporate resources.

## 1. Introduction

The field of gastronomic services is characterized by a strong competitive environment, and therefore, it is only natural that each of the market players is interested in bringing something new and innovative and thus in improving the experience for their customers. Customer expectations are formed already at the time of ordering food in a gastronomic facility. The second phase is made at the time when the food is served, and the person decides with all their senses what is his/her internal evaluation of the food or the restaurant in general [1]. The appearance and arrangement of the food on the plate are therefore decisive factors. Sight followed by smell are usually the first senses that make this decision for us [2].

One of the first theories presented was the claim that the method of serving and a certain arrangement of a particular food can, through our sight, affect our brain and subsequently our taste buds, and therefore, it can affect the overall experience of consumption [3]. People are attracted to beauty, and the same goes for the arrangement of food. Therefore, guests who receive food on a plate in style tend to claim that their food was tastier compared to guests who received the same food that was not very attractive on the plate [4]. Our eyes are connected through the brain to our mouths, and this fact is supported by the assumption that, if we like what we see, it is also tastier to us and we evaluate it better [5]. Many scientific studies in the subject matter were carried out in artificial laboratory conditions, where respondents often evaluated only pictures of food, which is incomparable with the evaluation of real food. Only a few studies were realized in real conditions of gastronomic facilities such as the canteen of Oxford University [6]. The research confirmed that food (appetizer) was perceived more stylish when served in a new way compared to the traditional style of serving, and guests were also willing to pay more for this style of serving, as it pleased their eyes more.

Consumer decision-making is influenced by a complex set of emotions, feelings, attitudes, and values, which cannot be assessed by classical feedback, e.g., by questioning. Recently, there has been a growing interest in the use of neuroscientific techniques to study consumer behavior. These make it possible to measure the emotional and spontaneous reactions of consumers more objectively [7]. The connection of neuroscience with gastronomy created another concept: neurogastronomy. It builds on the old term gastronomy and adds the adjective neuro to it, which refers to the brain. Neuroscientist Gordon M. Shephard discussed the definition and announcement of this new discipline in his book Neurogastronomy, which sought to summarize neuroscientific research on the perception and processing of tastes in the brain [8].

The modern field of consumer neuroscience or neuromarketing uses neuroscience to reveal subconscious consumer decision-making processes [9]. Consumer neuroscience uses both psychological and neuroscience methods to investigate marketing-related issues concerning buying behavior, thus offering a scientific explanation for consumer’s preferences and behaviors [10]. The neuroscience toolbox has many great options for exploring consumer experience, from measuring skin temperature and heart rate to more advanced technologies such as electroencephalogram (EEG) and functional magnetic resonance imaging (fMRI) [11]. Accordingly, it is possible to divide the research tools and techniques of neuromarketing into two main categories: biometric measurement (measuring the reactions of the body) and brain measurement (measuring the response of the brain) under the influence of marketing stimuli. Each approach captures different types of signal, and each brings a number of advantages and disadvantages depending on the used measurement technique [12].

There are several studies that have used either biometric or neuroimaging methods in conjunction with gastronomy. For example, the biometric eye-tracking method was used to monitor eye movement to detect the visual attention gathered by foods depending on the type of background [13] or to determine whether the smell or odors can increase attention via visually presented objects that correspond to a given scent [14]. Another biometric method, galvanic skin response (GSR), served to identify patterns of physiological activity during food selection in a virtual-reality buffet setting and a real-life buffet setting [15]. A practical example of the use of biometric (skin galvanic resistance and eye-tracking) and neuroimaging methods (electroencephalography) in combination with the technique of qualitative research (in-depth interview) is the empirical study in [16], which was conducted in a Michelin-starred restaurant. The analyzed variables were the presentation of the waiter or chef, the design of the plate, the food served, the taste of the food, the interaction, and the moment at which the food was served.

Neuroimaging method positron emission tomography (PET) was used to provide evidence that food presentation increases metabolism throughout the brain cortices [17], and the EEG technique demonstrated the effects of wine flavors on consumer preferences [18]. Another example of the use of a neuroimaging method, specifically functional magnetic resonance (fMRI) to verify how food presentation affects the consumer’s brain is the work of [19], and a series of fMRI scans of participants while viewing color pictures of food was collected by Wolfe et al. [20].

All these facts led us to our research and thus to testing the visual presentation of food using selected techniques of neuromarketing research. The main goal of the paper is to find out what effect the visual presentation of food prepared from the same ingredients and presented in three different ways has on consumer’s preferences. We developed the following research question: There are differences in the emotional perception of the same food served in three different ways of design. In order to reveal real preferences, not only conscious but also unconscious feedback of the respondents were studied using a mobile eye camera and a biometric method of recognizing emotions based on observable changes on the face—face-reading. A similar study design using a mobile eye camera was used to investigate the relationship between modifying the same food in three different ways and consumer preferences [21] or how suboptimal food shape can influence decision-making and consumer choice [22].

## 2. Materials and Methods

**Figure 1:**
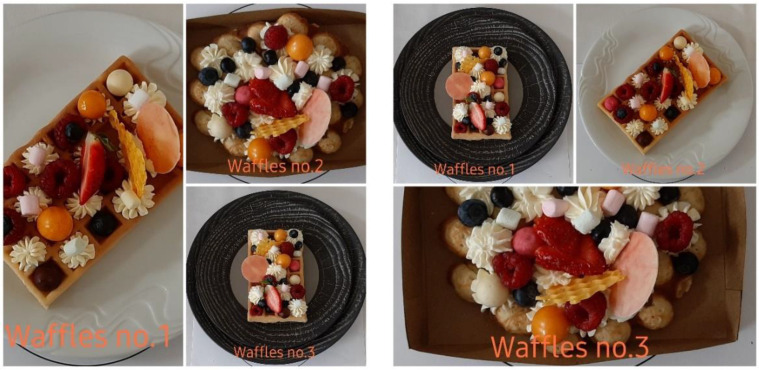
Testing variants of individual visual designs.

**Figure 2:**
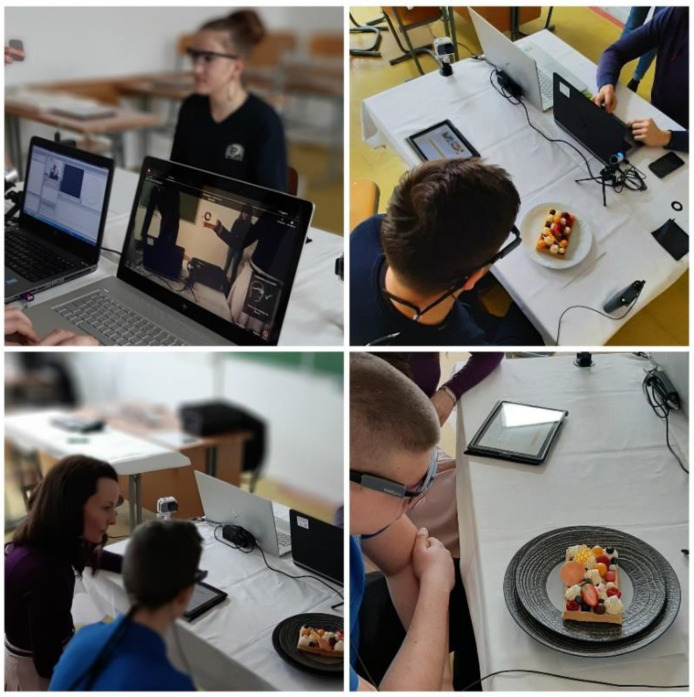
The course of testing the visual design of waffles.

The object of the research was food—waffles prepared and served in three different ways in cooperation with the Ľudovít Winter Hotel Academy in Piešťany. The first variant was served on a classic white plate, the second was served in the style of street food, and the third was served on a luxuriously designed black stone. The same ingredients and supplements were used to prepare all three meals to ensure the same look. Respondents evaluated the prepared samples on a conscious but also unconscious level in two rows to avoid distortion of the results. For the first sample in a row, the Latin squares method [23] was used; see Figure 1. In addition to the conscious and unconscious evaluation of the visual performance of the same food, the perceptual effect on the olfactory perception of the smell of sweet food was also observed; see Figure 2. The subjects in monitoring were also environmental factors (lighting, noise, and air quality), which fundamentally affect the emotions of the consumer, mostly on an unconscious level.

A total of 35 respondents took part in the testing. Due to problems with the initial calibration and incorrect recording of eye movements (less than 50% of the acquisition rate), we excluded 7 participants. The subjects of analysis were 28 healthy high school students aged 16–20 years. The condition for participation in the testing was the consumption of sweet foods. The size of our sample is comparable to similar neuromarketing studies using eye tracking, in which a sample ranging from 6 to 30 respondents was used with respect to international standards [24]. Of course, each survey is unique in its own way and the size and selection of the sample of respondents must be adapted accordingly. However, it is usually not recommended to analyze samples with less than 20 participants [25,26]. A similar sample of 32 respondents was used to test the effect of gaze direction on food preferences in [27].

The whole testing process was governed by the Code of Ethics “Laboratory of Consumer Studies” of the Faculty of Economics and Management of the Slovak University of Agriculture in Nitra [28] and by the neuromarketing science and business association (NMSBA). The NMSBA Code of Ethics for the Application of Consumer Neurosciences in Business [29] is the first step towards international standards for the use of neuroscience methods, while accepting the principles stipulated in the ICC/ESOMAR Code of Ethics (2020). The Code of Ethics “FEM SUA Consumer Studies Laboratory” for conducting research in the field of marketing, management, and economics using biometric and neuroimaging devices sets out the basic principles that must be followed by all those who carry out marketing research or use the results from it. This code of ethics is also in accordance with the International Code ICC/ESOMAR (Code of Ethics of the Laboratory of Consumer Studies FEM SUA Nitra, 2020). Each of the respondents was acquainted with the course of the experiment, completed a short training on the methods used (eye tracking and face reading) and filled in two forms in accordance with the GDPR, namely consent to biometric testing, processing, and storage of personal data and confirmation of short training.

Ensuring the quality of eye movement data was based on adherence to several aspects, including the suitability of the equipment used [30], equipment settings, experimental procedure, and the behavior and psychology of participants [31,32,33].

**Figure 3:**
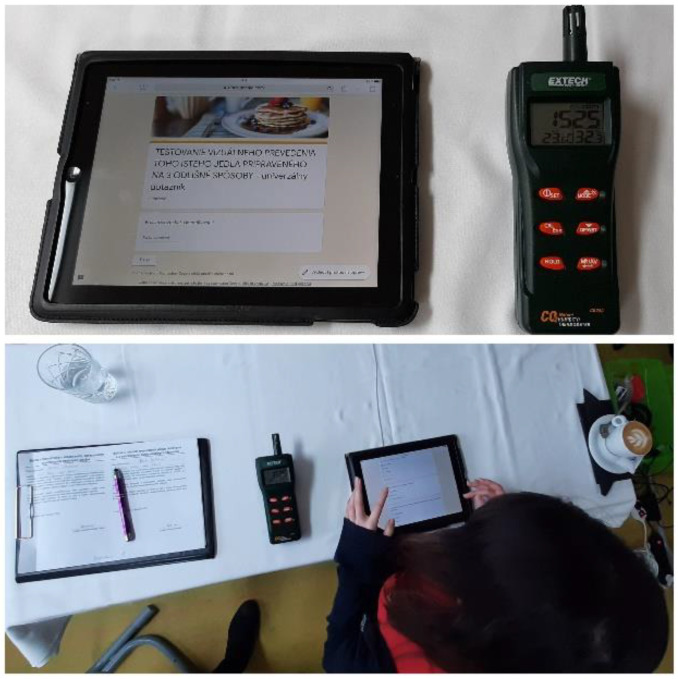
Introductory interview and monitoring of indoor air quality.

Subsequently, an initial interview and calibration of the instruments took place (see Figure 3). This is the stage where the experimenter has the greatest control over the quality of the eye tracking data, and extra care was taken to ensure that the calibration achieved acceptable levels of accuracy and precision [34]. After calibration, the respondents were gradually served meals in the specified order with a 15 s minimum looking-before-tasting phase. In order to eliminate manipulation of the results, recalibration was performed between respondents who wore glasses and lenses between serving individual meals [35,36]. The determination of the time interval was based on other studies [37] looking at the attention range of the participants. The subject of interest during the first 15 s was the monitoring of visual attention and emotional response evoked by the influence of the served waffle. Subsequently, the respondent evaluated his/her preferences at a conscious level and answered the questionnaire. The questionnaire was divided into three parts. The first two parts concerned the visual design and the smell of the tested food—waffles. During each meal, respondents answered five questions. The third part of the questionnaire focused on the general consumer behavior of waffle preferences, where three questions were answered.

**Figure 4:**
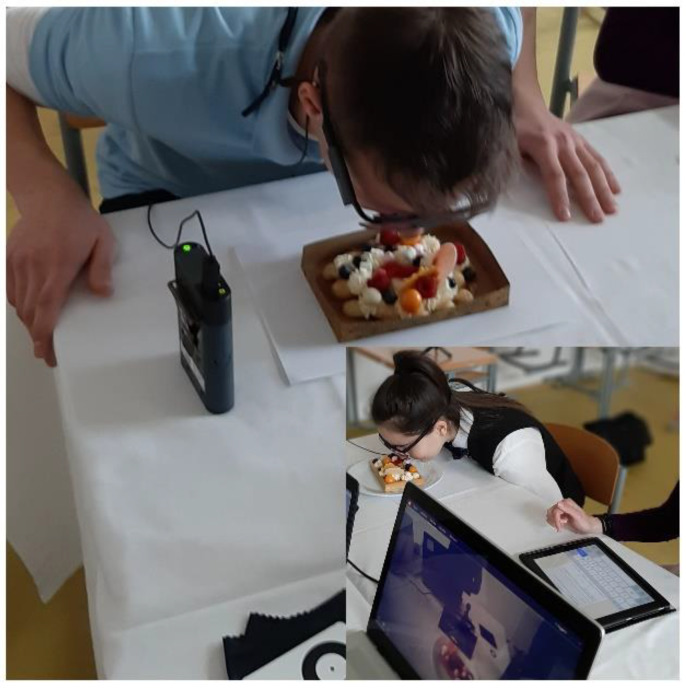
Evaluation of the aroma of individual visual designs of waffles.

A part of the testing was also to assess the smell of the visual design of the food in order to determine whether visual perception can affect the olfactory perception; see Figure 4. Even between olfactory and visual functions, there is a close connection, which is supported by neuroimaging studies demonstrating activation of the visual cortex during the performance of purely olfactory tasks [38]. We tried to incorporate scent into our research also because many published studies follow only the influence of one visual characteristic on the perception of tastes and consumer preferences. However, little is known about the complex interactions between more than two sensory modalities [39]. Knowing the effect of scent on our daily eating habits is essential. Namely, if we understand how and under what circumstances olfactory signals lead to the choice of foods/meals, this can have practical consequences, e.g., in using them to guide people to healthier eating [40].

The perception of tastes is one of the most complex manifestations of human behavior. It includes almost all the senses, especially the sense of smell, which is involved in the odor images generated in the olfactory path [41]. Smell and taste work closely together in determining food perception and taste, but their respective roles in taste, selection, intake, and satiety are very different. In particular, fragrances play a predictable role in stimulating taste in consumer behavior, as they are able to generate an appetite specific to a given product and, depending on other external or internal factors, also influence food preferences, their selection, and intake. However, it is clear that the relationship between olfactory signals and eating behavior is complex and means that odor stimuli evoke more than just affection or appetite [40].

In a 40 m^2^ room, the environmental factors (lighting, noise, and air quality) were also monitored during the test to ensure constant conditions. The most difficult was to ensure constant conditions of air quality, which was monitored by means of an Extech CO2 meter + humidity/thermometer. We also considered information about the weather [42,43], which can also affect the emotions of the participants. The controlled environmental factors can be seen in Table 1.

Emotions are considered important factors in food-related perception and behavior. However, a complicating factor is the fact that human emotions are a multifaceted construct associated with physiological, behavioral, and cognitive processes. Many types of measurements have been developed to evaluate food-induced emotions. However, most of them are based on subjective evaluations (conscious feedback). However, it should be borne in mind that, when individuals are offered food stimuli, they perceive and integrate information from all senses, and therefore, unconscious processes gain importance. Thus, either physiological measurements or measurements reflecting brain activity can be used to evaluate them [44].

In our case, measurements based on the body’s response were used. In this case, special glasses were used to monitor eye movements—mobile Eye tracker glasses 2 by the Tobii Company (Danderyd, Sweden). This device uses the Pupil Centred Corneal Reflection—Dark pupil eye-tracking technique, which is a binocular system focusing on both eyes with a sampling frequency of 120 Hz [45]. Primary data recorded by this method were processed in the Tobii Pro Lab software environment version 1.83.11324 [46].

Emotional feedback was monitored using the somatic biometric method Facereader 7 from the Dutch company Noldus (Wageningen, the Netherlands), which identifies the emotional feedback (valence and excitement) of respondents with maximum accuracy based on observable changes in mimic muscles and recognizes basic micro-emotions (happy, sad, angry, disgusted, surprised, and neutral) [47]. The validity of the recorded data was mainly influenced by the scanning angle, the brightness of the environment, and the resolution of the recording device [48].

The validity of eye data due to visual stimuli depends, in addition to the abovementioned precise and uniform instructions for respondents, also on several details [15] such as complexity and diversity [49,50], stimulus size, and quality [51]. In our case, unlike most studies, we used real food stimulus and the same weight for all three visual versions: 150 g.

At the same time, the quality of the data obtained was influenced by a number of cognitive factors knowledge, expectations, significance/value for the respondent [52,53], and affective factors [54,55] partially eliminated by informing about the course of testing and the sample.

The data obtained from individual measurements were synchronized and mutually correlated in the Observer XT 10 software environment from Noldus. This program allows you to synchronize structured and unstructured data from individual devices and, at the same time, to create your own variables during the implementation of experiments [56]. Descriptive and inductive statistics methods were used to process the primary data.

Processing took place in a programmatic environment:

- Matlab R2019a,
- Mathematical-statistical program R,
- Microsoft excel 2010.

The Wilcoxon paired test was used for inductive statistics.

## 3. Results

**Figure 5:**
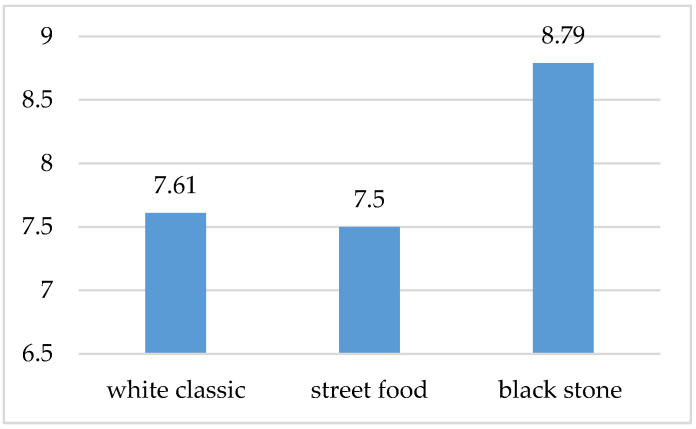
Conscious evaluation of the visual presentation of waffles served in three different ways.

Based on the initial interview, all participants in the survey stated that they sometimes consume the given food (waffles); 50% of them described the visual design of waffles as important, and 40% of them described it as a very important aspect of decision-making, while only 3 respondents could not take a stand and answer. From the participating group, 64% of people stated that they also visit gastronomic facilities in order to consume this type of food. According to the results based on an interview with test participants (see Figure 5), it can be stated that the respondents most positively rated the visual presentation number three based on conscious perception, which represented a waffle arranged on a luxurious black plate (arithmetic average based on points 8.79).

In this type of visual design (black stone), the respondents most often stated that they liked the arranged fruit (10) and the luxury plate (8), while they also mentioned the fruit as an element of “liking” in the other two visual designs. As can be seen from the visual on a classic white plate, the respondents also mentioned the most common fruits (11) and color (7). On the contrary, in the case of the street food version, the respondents liked most the box (7), the fruit (6), and the shape of the waffle itself (6).

**Figure 6:**
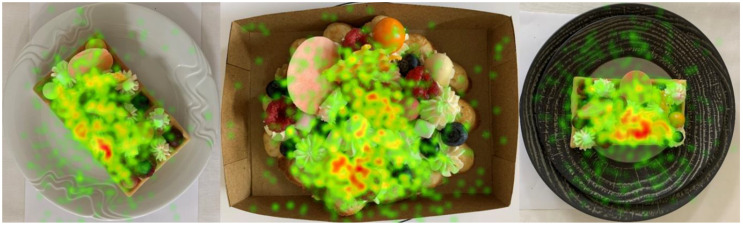
Thermal map of visual attention of waffles served in three different ways.

The results of the measurement of visual attention (Figure 6) showed that the respondents looked at the visual designs of the waffles on a classic white plate and the street food version for a demonstrably longer time. The duration of looking at individual ingredients or elements on waffles is interesting. While the respondents mostly looked at the strawberry on the street food version (on average 3.09 s), on the first version, it was white chocolate (on average 1.94 s) and an imitation of a honeycomb (on average 1.85 s).

**Figure 7:**
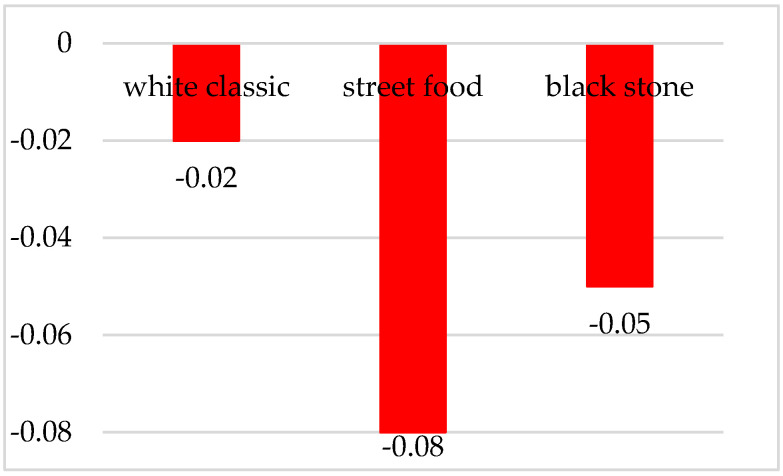
Level of excitement during the evaluation of waffles served in three different ways.

In terms of emotional response (Figure 7), the highest degree of arousal was recorded for the black stone meal presentation at a score of 0.377, which may have caused the luxurious appearance of the meal.

**Figure 8:**
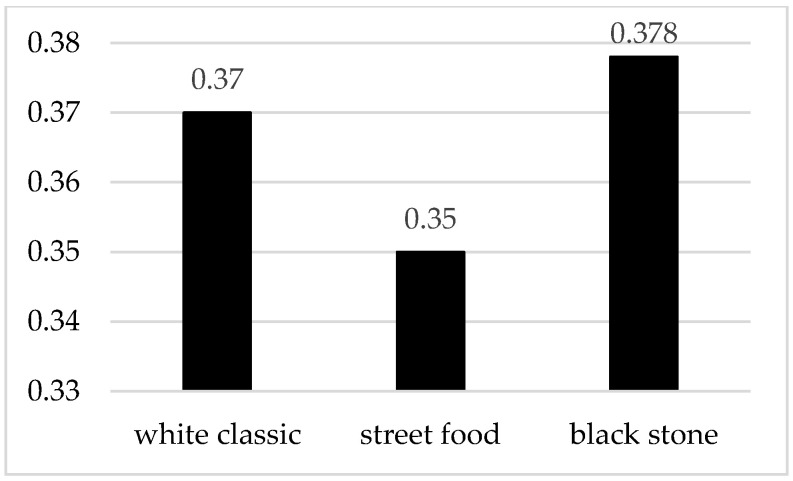
Valence during the evaluation of waffles served in three different ways.

Based on the average values, the most positive feelings in terms of testing visual presentations of food were waffles on a classic white plate with a score value of −0.02, which can be considered a neutral response. This may have been due to the fact that respondents did not consider the food to be too expensive and at the same time too cheap. Also, in terms of visual attention, this arrangement attracted them the most. On the contrary, based on an average of −0.08, the respondents rated the street food presentation the most negatively; see Figure 8.

We also tested the difference in emotions using the Wilcoxon paired test (Table 2), which found that there were significant differences in the emotions of the tested participants between visual 3 and visual 2 and between visuals 1 and 2. On the contrary, differences were not confirmed between visuals 1 and 3.

**Figure 9:**
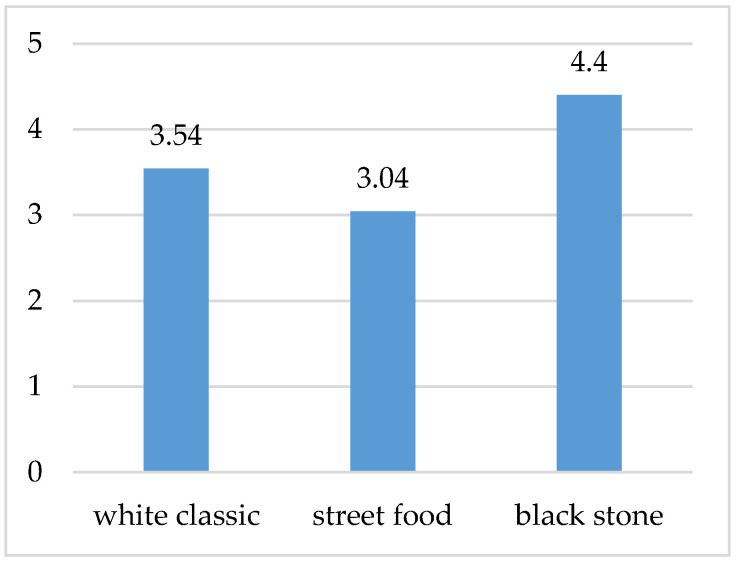
Willingness of respondents to pay for individual waffle designs (average in EUR).

These results, through a statistical test, suggest that creativity in arranging food is important not only as far as the color and complexity of the individual components are concerned but also taking into account the tray itself (plate and box), which together form one visual whole. It is known that a suitably aesthetically arranged final meal is evaluated by the guests as tastier or more valuable, which was also reflected in our survey; see Figure 9.

The respondent would be willing to pay an average of EUR 4.4 for a waffle on a stone plate, EUR 3.54 for that on a white classic plate, and EUR 3.04 for that as street food. The difference between the street food visual and the stone plate is almost 0.90 EUR, which indicates a significant impact on the perception of value in terms of arranging and serving food. It is also interesting that the street food visual was of lower value for the respondents (they were willing to pay less on average), despite the fact that the preparation of this visual represents higher unit costs than, e.g., serving on a classic plate (disposable street food box).

**Figure 10:**
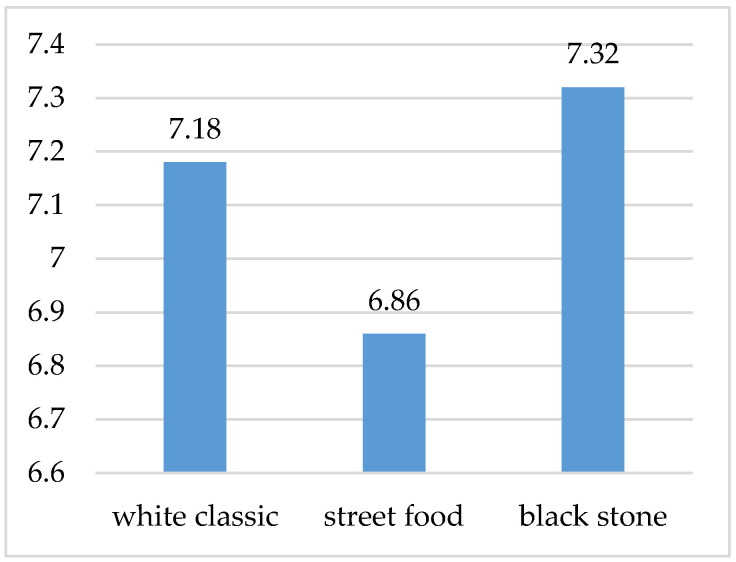
Evaluation of aromas of individual waffle variants.

We also tried to prove the influence of visuals on the different perceptions through the perception of scents from the individual variants. For this reason, we also focused on the smell of the waffles during the study, which was rated on a scale from 0 (least fragrant) to 10 (most fragrant). As in the case of the visual design of waffles, the waffles on a black stone smelled best to the participants (average rating 7.32), then the waffles on a white plate (7.18), and the least significant smell was recorded in the case of street food waffles (Figure 10).

It follows from the above that the visual design of the food and the method of serving can affect the perception of the smell of the food. The fact is that the taste and olfactory sense organs are interconnected and, therefore, smell is an integral part of food. Researchers found that scent enhances a product’s distinctiveness. The subjects remembered the scented product to a much higher degree than the unscented goods. The human brain processes first-time smells in a different way than familiar ones. The special processing, which can associate the smell with a pleasant or unpleasant experience, is unique to our sense of smell [57].

**Figure 11:**
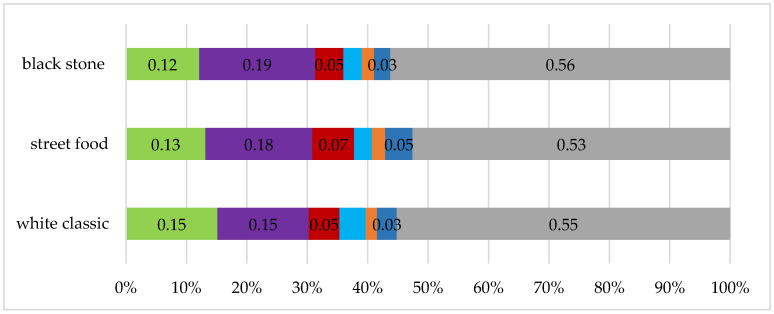
Microemotions recorded during the evaluation of individual visual versions of the food.

In terms of recorded microemotions (Figure 11), while looking at the visuals of individual waffle designs, neutral feelings prevailed (black stone 56%, street food 53%, and white classic 55%), while feelings of joy were also recorded (black stone 12%, street food 13%, and white classic 15%), grief (black stone 19%, street food 18%, and white classic 15%) and anger (black stone 5%, street food 7%, and white classic 5%), with the result that, based on the average, the overall perceived visual designs are neutral with the exception of the street food version (valence −0.08).

**Figure 12:**
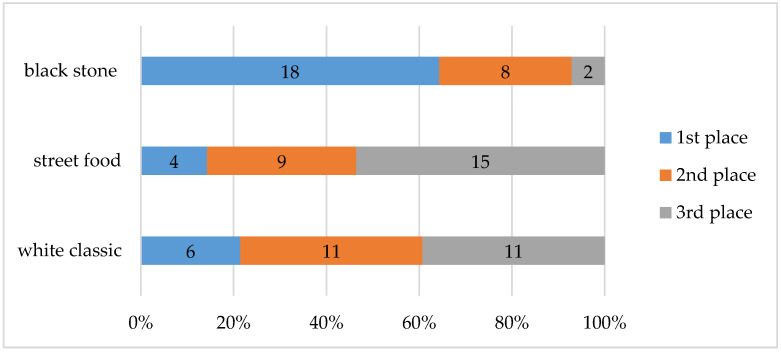
Overall evaluation of individual variants of waffles served in different ways.

In the overall evaluation of individual visual performances by arranging them in order, the respondents most often listed waffles prepared on a luxurious black plate in first place (64%). On the contrary, most respondents mentioned street food style waffles in third place (54%). In the case of food served on a classic white plate, the respondents, to a large extent, stated second place (39%) but also third place (39%); see Figure 12.

## 4. Discussion

Sight provides the primary sensory input for food perception. It raises expectations regarding taste and promotes acceptance or rejection of food [58]. Food that is served attractively appears to be tastier [59]. Creativity in arranging food builds not only on the color and complexity of the individual components but also on the tray itself (plate and box), which together form one visual whole. It is known that the guests not only evaluate appropriately aesthetically arranged final food as tastier but also are willing to pay more for it, which was also reflected in our survey. The difference between the street food visual and the stone plate is EUR 1.36, which indicates a significant impact on the perception of value in terms of arranging and serving food. Our results are in some respects comparable to other studies [6,60], which also concluded that consumers perceive food tastier and are willing to pay more for it when served in an attractive way. At the same time, our research confirmed that the visual design of the food and the way it is served can influence the perception of the smell of the food. The fact is that the taste and olfactory sense organs are interconnected, and therefore, smell is an integral part of food.

Exploring the visual perception of desserts served in different ways has been the subject of research by other authors. The authors of [1] in their study compared the perception of color and contrast of food with its background, i.e., the background of the plate. They focused on strawberry mousse and served it on two color backgrounds—lighter/white and darker/black. The main focus was to examine changes in the intensity of taste, liking, and sweetness. The pink strawberry mousse was rated as more intense, sweeter, and tastier to the participants when served on a white plate than when served on a dark plate. Interestingly, only the color of the plate was important, not its shape. The different shapes of the plate—triangular, square, or round—did not cause any significant changes in the overall perception of the aspects that the study focused on. The authors of [61] went even deeper and tried to find out whether the color and shape aspects of the plate are important for taste perception. They experimented with the attributes of quality, intensity, sweetness, and taste in an experiment with cheesecake. They found that there was a correlation between the color and shape of the plate and the perception of taste, but other variables also needed to be taken into account. Another study [62] also confirmed the importance of the color of the plates, i.e., the background color with respect to the expectations and perception of food by consumers. Guests who took part in the research in the real environment of the restaurant received three desserts on a white or black plate. The results showed that the background color affected the attributes based on visual elements and the taste attributes depended on the type of the desserts served. Regardless of the results of each experiment, the finding is that the shape and color of the plate in interaction can affect the perception of tastes.

It is necessary to mention in this context the concept of “food design”, which represents a relatively new concept so far with insufficient academic research in this area [63]. The definition of food design given by [64] suggests that one of its main goals is to evoke emotions and expectations and to experience emotions [65]. This difference in the design of the same food served in three different ways was also proven in our survey. In general, visual attention reflects mental processing of the current view [66], which is also related to some form of affective perception presented in our survey by the differences in the thermal maps of the views. In addition, in our study, we also recorded a comparison of the emotional response based on visible facial expressions, which allows us to capture rapidly changing emotions and a subconscious part of the experience [67] from a given stimulus. In this case, they represented statistically significant differences in the polarity of emotions (valence) due to the visual designs of the waffles.

During our testing, we also discovered several shortcomings; therefore, we plan to carry out similar research in the future, in which we will pay much more attention to the size of plates, i.e., we will make them the same. In the current research, the size of the plates used was different, which could affect the visual perception of the food [51]. At the same time, during further testing, it will be necessary to ensure that the food served is always placed in the same way in front of the respondent (vertically or horizontally). The tested foods must be completely identical and equally decorated [15]. In the case of street food, a different shape for the waffle was used than in the other two samples, and the individual ingredients were not exactly the same style. We also plan to expand in-depth interview questions that map respondents’ tastes to specific visuals, which requires the selection of appropriate sensory analysis methods and the development of an experimental concept prior to data collection [68].

Despite the shortcomings, this contribution can be considered beneficial, as there are few studies using biometric methods to test the visual presentation of food, including consideration of environmental factors [69].

Proof of this is that the application of neuromarketing techniques in the food industry has recently gained considerable popularity in academia and commerce, for example large research companies such as Nielsen, Kantar, or Ipsos, which have included neuromarketing tools in their commercial offerings [70]. Despite many critical aspects, such as questioning the privacy limit or the concept of free will, the incorporation of these technologies and consumer behavior and market research is an important part of understanding and meeting research goals today [71].

Several aspects affect the consumer’s decision-making and choice, including mood or emotional state [72,73]. The role of emotions in the consumer decision-making process is explained by the principle of neurological and cognitive frameworks, such as the theory of somatic markers [74], which focuses on the so-called attention given to the negative effects of decision-making.

The development of neuromarketing discipline in the food industry also faces some concerns about the interpretation and findings of some commercial studies to date [75], given that some companies even make controversial claims without evidence-based citations [26,76]. In this context, it should be borne in mind that academic studies are based on strict protocols and adherence to methodological procedures [77], which allows for a new perspective on unconscious consumer perception. Nevertheless, even in this context, there are some critical views on the existence of strong unconscious influences on decision-making and related behavior [78].

Hall tests, surveys, or observations are usually used to understand the perception of food products and food. However, these approaches require larger samples of respondents to obtain reliable results and are based on the assumption that participants are able to express their preferences [79]. It should not be forgotten, however, that food stimuli can affect preferences and eating habits also unknowingly [80], and therefore, the use of neuroscientific methods has its justification in this area. Their main goal is to measure crucial aspects of consumer perception not only on the unconscious (attention, emotional response, and memory) but also on the declarative levels (attitudes and preferences) [7,81], so that the findings can be applied in managerial decision-making in the creation of effective sales strategies [70].The use of a relatively small sample size may result in lower statistical power [82]; therefore, in the future, we plan to conduct research on larger samples of respondents, which would allow us to examine visual preferences depending on selected sociodemographic characters, such as [83] in their research.

At the same time, in our further measurements, we plan to incorporate environmental factors and weather information to a much greater extent than in the current research, which can also significantly affect consumer behavior and emotions, as the following authors have shown. The authors of [84] summarized the effects of weather moods followed: a lack of sun awakens sadness; a bright sun promotes wasting money; rain causes appetite, people to be more sensitive to pain, and staying outdoors and thus improving memory and promoting creativity. The authors of [85] present that only a negative affect mediates the effect of weather on spending (i.e., changes in positive affect do not impact spending). Bad weather keeps people at home. In particular, rain, snow, and extreme temperatures have been identified as factors that make go out to shop. When temperatures fall, ice cream sales decrease while sales of oatmeal porridge increase. The authors of [86] introduced that studies in psychological literature have examined in depth the relationship between mood or emotional states and weather, establishing an accepted link between weather variables such as temperature, humidity, sunshine hours, precipitation, barometric pressure, and wind velocity. Situational variables affect human propensity to buy, extending to the amount of time spent in a store and willingness to return. Nice weather has been found to correlate positively with self-assessments of mood and with tips left at restaurants [87].

Last but not least, we plan to extend this research to other methods of obtaining unconscious feedback, such as electroencephalography [88], which provide meaningful insights and predictive results even when using smaller samples [89,90]. Although a number of other critical artifacts associated with the use of neuromarketing tools need to be removed [91], this study is a starting point for further research that will provide several solutions to problems related to marketing management in the food and catering segment.

## 5. Conclusions

Important visual factors influencing the perception of the presented food include its distance from the consumer, variety, diversity, distribution on a plate, portion size, and the gastronomic service (plates) used. In cooperation with the Ľudovít Winter Hotel Academy in Piešťany, we carried out research to find out how people react to the same food (waffles) served in three different ways but using the same ingredients. The first version had a traditional rectangular waffle served on a white plate; the second was in the style of street food with a round waffle served in a rectangular cardboard box; and the last visual was served as a traditional rectangular waffle on a luxurious black stone plate. In all three cases, the same ingredients (fruit, whipped cream, candies, white chocolate, and imitation of honeycomb) were used for decoration without the use of any icing for practical reasons during all-day testing. The research involved 28 students aged 16 to 20 years (50% women and 50% men). All respondents stated that they sometimes consume the food in question (waffles).

The methodology of the experiment was designed so that each participant was brought a covered meal, which he/she looked at for about 15 s after uncovering it. Subsequently, he/she expressed his/her preference and impression through an electronic questionnaire form. The order in which the individual visual variants were presented has been changed to ensure maximum transparency of the recorded data. The Eye Tracker mobile eye camera and FaceReader emotional recognition software were used to obtain data on unconscious perception.

The best visual perception on a conscious level was found when the food was served on a black stone, the second was on a white classic plate, and the third was in the case of a street food box. Food consisting of rectangular ingredients as well as ingredients of smaller pieces evenly sprinkled on waffles was perceived by people as larger and visually more attractive compared to meals with round ingredients. If the food occupied a larger surface, it was eaten less because it created the impression of a larger volume (street food visual). At the unconscious level, the respondents perceived a waffle served on a classic plate the most positively (−0.02). The statistical test confirmed the different perceptions, especially in the style of arranging the street food in comparison with serving on plates. However, the level of excitement was the highest with a luxury stone plate (0.377), which coincides with the conscious feedback. The higher level of excitement in this case was probably caused by the luxurious appearance of the arranged food, which is also confirmed by the attribution of a higher value to this variant of visual design. Respondents were willing to pay an average of EUR 4.4 for a waffle on a stone plate, which is almost EUR 1.36 more compared to a street food version. The willingness to pay a higher price could have led to a higher appetite for the food served in this way, as the appetite is increased by the right color-balanced ingredients and the appropriate contrast of the food on the plate. Nevertheless, the results from measuring visual attention showed that the respondents looked demonstrably longer at the visual designs of waffles on a classic white plate and the street food version. The duration of looking at individual ingredients or elements on waffles is interesting. While the respondents mostly looked at the strawberry on the street food version (on average 3.09 s), on the first visual variant, it was white chocolate (on average 1.94 s) and an imitation of a honeycomb (on average 1.85 s). The visual design of the waffles also influenced the sensory perception, since in constant conditions in terms of air quality, the respondents were most pleased with the food prepared on a black stone plate (black stone 7.3).

It follows from the above that, in assessing the emotional response, in addition to the use of tools for classical feedback collection, it is important to reach for the measurement of subconscious reactions based on monitoring eye movements and facial expressions, facial biometrics, which provides a whole new perspective on the perception of the visual presentation and serving of food. Despite all the shortcomings, this contribution can be considered beneficial, as there are few studies using biometric methods to test the visual presentation of food, including consideration of environmental factors.

## Figures and Tables

**Table 1:** Controlled environmental and weather factors.

Element	Factor	Unit	Value Range	
Lighting	Intensity	Lux	280–330	
	Chromaticity temperature	K	3600–4000	
Noise	Intensity	dB	24–44	
Air quality	Temperature	°C	24–25	
	Humidity	%	43	
	CO_2_	Ppm	560–700	
**The Weather**				
**Temperature**	**Humidity**	**Precipitation (probability)**	**Character**	**Pressure**
4–5 °C	41%	0%	Sunny/clear	1016 hPa

**Table 2:** Wilcoxon paired test—comparison of valence of individual visuals.

	Visual 1	Visual 2	Visual 3
Visual 3	H0	H1	
Visual 2	H1		
Visual 1			

H0—no difference, H1—there is a difference, testing at α = 0.1.

## Data Availability

Data available on request due to restrictions, e.g., privacy or ethics.
